# Neutrophil proteome shifts over the myocardial infarction time continuum

**DOI:** 10.1007/s00395-019-0746-x

**Published:** 2019-08-15

**Authors:** Michael J. Daseke, Fritz M. Valerio, William J. Kalusche, Yonggang Ma, Kristine Y. DeLeon-Pennell, Merry L. Lindsey

**Affiliations:** 1 Department of Physiology and Biophysics, University of Mississippi Medical Center, Jackson, MS 39216 USA; 2 Department of Molecular Pharmacology and Physiology, University of South Florida, Tampa, FL 33612 USA; 3 Ralph H. Johnson Veterans Affairs Medical Center, Charleston, SC USA; 4 Division of Cardiology, Department of Medicine, Medical University of South Carolina, Charleston, SC 29425 USA; 5 Department of Cellular and Integrative Physiology, University of Nebraska Medical Center, 985850 Nebraska Medical Center, Omaha, NE 68198-5850 USA; 6 Research Service, Nebraska-Western Iowa Health Care System, Omaha, NE USA

**Keywords:** Myocardial infarction, Neutrophil, Proteomics, Aptamer, LV remodeling, Cell polarization

## Abstract

In response to myocardial infarction (MI), neutrophils (PMNs) are early responders that initiate the inflammatory reaction. Because macrophages and fibroblasts show polarization states after MI, we hypothesized PMNs also undergo phenotypic changes over the MI time course. The objective of the current study was to map the continuum of polarization phenotypes in cardiac neutrophils over the first week of MI. C57BL/6J male mice (3–6 months old) underwent permanent coronary artery ligation to induce MI, and PMNs were isolated from the infarct region at days 1, 3, 5, and 7 after MI. Day 0 served as a no MI negative control. Aptamer proteomics was performed on biological replicates (*n* = 10–12) for each time point. Day (D)1 MI neutrophils had a high degranulation profile with increased matrix metalloproteinase (MMP) activity. D3 MI neutrophil profiles showed upregulation of apoptosis and induction of extracellular matrix (ECM) organization. D5 MI neutrophils further increased their ECM reorganization profile. D7 MI neutrophils had a reparative signature that included expression of fibronectin, galectin-3, and fibrinogen to contribute to scar formation by stimulating ECM reorganization. Of note, fibronectin was a key modulator of degranulation, as it amplified MMP-9 release in the presence of an inflammatory stimulus. Our results indicate that neutrophils selectively degranulate over the MI time course, reflective of both their intrinsic protein profiles as well as the ECM environment in which they reside. MMPs, cathepsins, and ECM proteins were prominent neutrophil degranulation indicators.

**Electronic supplementary material:**

The online version of this article (10.1007/s00395-019-0746-x) contains supplementary material, which is available to authorized users.

## Introduction

Myocardial infarction (MI) initiates a cardiac wound-healing cascade that originates with myocyte necrosis to stimulate inflammation and leukocyte influx and culminates with scar formation. By 24 h after MI, the prominent leukocyte is the neutrophil [25]. While excess neutrophil influx is detrimental by promoting excessive tissue breakdown to enhance dilation of the left ventricle (LV), neutrophil depletion also amplifies inflammation and LV dilation to reduce cardiac performance in rodent models and humans [17, 18, 41]. Neutrophils, therefore, are essential for the wound-healing process.

Neutrophils undergo degranulation, releasing proteases to degrade the extracellular matrix (ECM) and facilitate leukocyte infiltration [13, 42]. The Steffens laboratory reported that neutrophils also orchestrate MI healing by polarizing macrophages toward a reparative phenotype, assigning an indirect role for neutrophils in LV remodeling [18]. Our laboratory observed that neutrophils themselves undergo polarization after MI, with > 95% of day 1 neutrophils expressing N1 markers (Ccl3, Ccl5, and Tnfα) and 20% of day 7 neutrophils expressing N2 markers (Tgfβ1, Il10, and Cd206) [26]. N1 neutrophils activated by damage-associated molecular patterns (DAMPs) link to an early increase in MI wall thinning. Neutrophils, therefore, have roles in both promoting and turning off inflammation.

The current study extends past reports by mapping neutrophil transitions in response to MI. We analyzed proteomic changes at MI days 1, 3, 5, and 7 to reflect the early inflammatory, proliferative, and maturation phases compared to day 0 no MI neutrophils. We hypothesized that neutrophils would undergo phenotypic changes over the MI time course that range from pro-inflammatory to reparative polarization. We used an aptamer proteomics approach, because mature neutrophils are post-mitotic and have reduced transcriptional capacity compared to dividing cells [15, 34, 43]. To our knowledge, this is the first study to report in detail the full proteome changes that occur in cardiac neutrophils that mediate post-MI wound healing and remodeling.

## Methods

### Animal use, coronary artery ligation, echocardiography, and necropsy

Detailed methods are provided in the Supplemental Methods [5, 21, 23]. Because neutrophils exhibit time of day variation, with MI surgery performed in the evening resulting in higher rupture rates due to higher neutrophil recruitment, all surgeries were performed between 8 am and noon [40].

### Isolation of LV infarct neutrophils

LV neutrophils were isolated from the combined infarct and border region by immunomagnetic separation (Supplemental Methods) [10, 19, 31]. The neutrophils obtained by this method are highly (> 99%) pure [26].

### Protein isolation

Neutrophil cell pellets were lysed in Reagent 4 (Sigma C0356; 50 μL per 1 × 10^6^ cells) with 1× protease inhibitor cocktail (Roche), centrifuged at 14,000×*g* for 5 min, and the supernatant was collected. Total protein was quantified using the Bradford assay. Sample concentrations were adjusted to 200 μg/mL using phosphate-buffered saline (PBS) and 100 μL of sample was sent to SomaLogic (Boulder, CO) for aptamer proteomic evaluation.

### Aptamer proteomics

Aptamer proteomics uses short single-stranded DNA sequences that bind to proteins. The SOMAscan^®^ multiplex aptamer-based proteomics platform on a custom Agilent hybridization chip was used to analyze 1305 proteins (assay version 3.2) [14, 20, 30, 33, 44]. Aptamers are chemically modified unique and single-stranded DNA segments that bind to specific proteins. Data normalization and calibration were performed according to the SOMAscan^®^ Data Standardization technical note (SSM-071) [7].

### In situ hybridization and immunohistochemistry multiplex imaging

In situ hybridization and immunoblotting histological evaluations were performed according to the guidelines for authors and reviewers on antibody use in physiology studies (Supplemental Methods) [6].

### Isolation and stimulation of bone marrow-derived neutrophils

Bone marrow-derived neutrophils were isolated from the tibias and femurs of control no MI mice under isoflurane anesthesia as previously described [26]. Ly6G + neutrophils were diluted to 2 × 10^6^ cells/mL in RPMI 1640 media with 1% antibiotic solution. Cells (1 × 10^6^) were unstimulated or stimulated with the positive control phorbol 12-myristate 13-acetate (PMA; 20 nM) or fibronectin (100 ng/mL) for 15 min at 37 °C. The cells were centrifuged at 800×*g* for 10 min, and the supernatant (200 μL) was analyzed using the Proteome Profiler Mouse XL Cytokine Array (R&D Systems, ARY028).

### Statistics and bioinformatic analyses of the proteomics dataset

Statistical analyses were performed according to established guidelines [22]. All experiments were performed and analyzed in a blinded design, and data are presented as mean ± SEM unless otherwise noted. For echocardiography, comparisons were made using one-way ANOVA followed by Newman–Keuls post hoc test. A value of *p* < 0.05 was considered statistically significant.

Aptamer proteomics results are given as relative fluorescence units (RFUs). Prior to bioinformatics analysis, a quality control assessment was performed (Supplemental Methods) and the data log transformed for normalization. Analyses tools available in the online resource MetaboAnalyst 3.0 (http://www.metaboanalyst.ca/) and GraphPad Prism were used for statistical and bioinformatics analyses [47, 48]. One-way ANOVA with Tukey’s post hoc test was performed to determine differentially expressed proteins using a false discovery rate (FDR) adjusted *p* value cutoff of 0.05. For individual MI days, differential expression was characterized by a fold-change threshold of > 2.0 or < 0.5 compared to day 0 no MI values and a *p* value of less than 0.05 by two-tailed unpaired *t* test. Proteins were ranked first by *p* value and then by fold change.

Enrichment analysis for differentially expressed proteins was performed using Enrichr (http://amp.pharm.msm.edu/Enrichr/) gene ontology (GO) biological processes and Ingenuity Pathway Analysis (Qiagen) canonical pathways. For GO terms, the combined score (calculated from *Z* score and *p* value) was reported. Proteomic data comparisons to galectin-3 and fibronectin immunoblotting were made by one-phase association analysis to obtain goodness of fit *R*^2^ values.

## Results

### MI confirmation

**Fig. 1:**
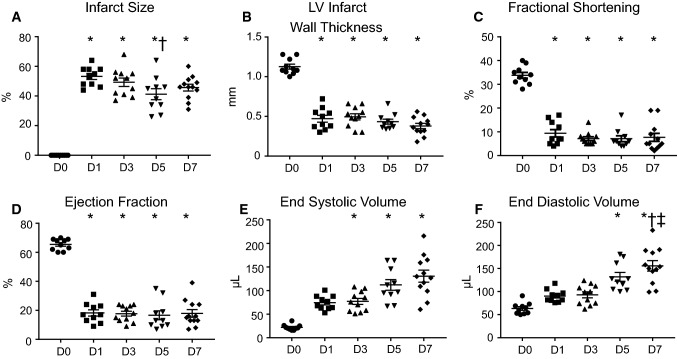
Proof of myocardial infarction (MI) by coronary artery ligation. Measurements were taken at day (D) 0, 1, 3, 5, and 7 after MI. **a** Infarct size (infarct weight as a percent of total LV weight). **d** Left ventricular infarct wall thickness (mm). **c** Fractional shortening (percent). **d** Ejection fraction (percent). **e** End systolic volume (µL). **f** End diastolic volume (µL). *p ≤ 0.05 vs. day 0, ^†^*p* < 0.05 vs. day 1, and ^‡^*p* < 0.05 vs. day 3 by one-way ANOVA followed by Newman–Keuls post hoc test, sample sizes are *n* = 10 day 0, *n* = 10 day 1, *n* = 11 day 3, *n* = 10 day 5, and *n* = 12 day 7

All MI times showed robust infarct areas (range 40–60% of LV area; Fig. 1a). Infarct wall thickness (Fig. 1b), fractional shortening (Fig. 1c), and ejection fraction (Fig. 1d) decreased at D1 through 7 MI, and LV volumes (Fig. 1e, f) increased at D3 through D7 MI.

### Neutrophils differentially polarize from MI days 1–7

**Fig. 2:**
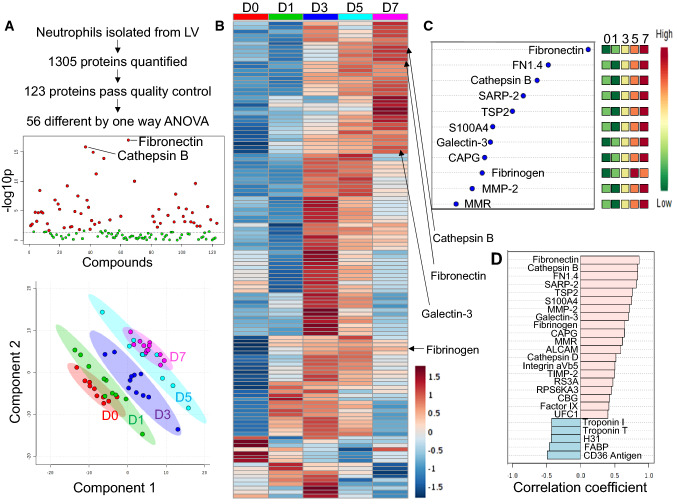
Proteomic analysis in neutrophils revealed a phenotypic shift over the MI time continuum. **a** By one-way ANOVA, 56 proteins were different (top). Graphical representation of partial least squares discriminant analysis (PLS-DA; bottom). **b** Heat map comparing protein expression across days 0–7. Each bar represents the average of *n* = 10–12 mice. **c** Important feature analysis indicated fibronectin was the strongest feature over the entire MI time course. **d** Correlation analysis also showed fibronectin as the strongest protein correlating with MI day

Of the 123 proteins that passed quality control (Supplemental Table 1), 56 proteins were statistically different by ANOVA (Fig. 2a top and Supplemental Table 2). Partial least squares discriminant analysis (PLS-DA) revealed distinct neutrophil protein expression patterns across MI day, indicating a temporal change in polarization status (Fig. 2a bottom). Patterns of individual protein changes over MI time were visualized in a heat map (Fig. 2b). Important feature analysis (Fig. 2c) and correlation analysis (Fig. 2d) indicated that the ECM protein fibronectin continually increased with MI time. Of note, mannose macrophage receptor (MMR; Cd206) showed a linear increase with MI time, consistent with our past report [26].

### MI D1 neutrophil polarization phenotype: pro-inflammation, degranulation, and invasion

**Fig. 3:**
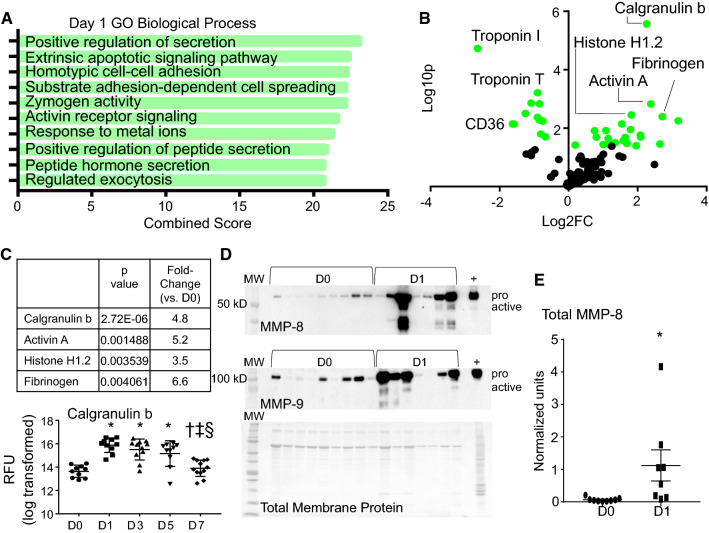
Neutrophil expression profile at day 1 post-MI. **a** Gene ontology (GO) biological processes enriched in day 1 MI neutrophils. **b** Volcano plot analysis, with downregulated proteins on the left and upregulated proteins on the right. Proteins with *p* values < 0.05 are shown in green. **c** Calgranulin b and IL-16 were upregulated at MI days 1 and 3. *p < 0.05 vs. D0, ^†^*p* < 0.05 vs. D1, ^‡^*p* < 0.05 vs. D3, and ^§^*p* < 0.05 vs. D5; and sample sizes are *n* = 10 day 0, *n* = 10 day 1, *n* = 11 day 3, *n* = 10 day 5, and *n* = 12 day 7. **d** MMP-8 and MMP-9 immunoblotting showed strong upregulation at MI day 1 (total membrane protein stain at the bottom). **e** Quantification of MMP-8 immunoblotting. *p < 0.05, sample sizes are *n* = 9 for day 0 and 8 for MI day 1

By GO biological process evaluation, exocytosis and pathways involved in secretion (degranulation) were the most enriched (Fig. 3a). Calgranulin B (S100A9), activin A, histone H1.2, and fibrinogen were the highest ranked changes, by *p* value and fold change compared to D0 (Fig. 3b). The decrease in troponin I and T as well as CD36 within the neutrophil fraction may represent baseline D0 phagocytosis of myocyte components that reflect a previously unknown surveillance role for the resident cardiac neutrophil. We have previously reported that about 5% of resident cardiac cells are neutrophils [26].

In a separate Kyoto Encyclopedia of Genes and Genomes (KEGG) enrichment analysis, the top three enriched pathways in MI D1 neutrophils were complement and coagulation cascades (combined score 20.56), platelet activation (combined score 18.84), and cytokine–cytokine receptor interaction (combined score 14.04; all *p* < 0.001). This indicates that MI D1 neutrophils are predominantly pro-inflammatory. In acute inflammation, neutrophils and platelets are simultaneously activated to regulate the inflammatory response. Heteromers of proteins contributed from both cells form to promote monocyte recruitment [1], and our results indicate that this happens beginning at day 1. Neutrophils interact with platelets by both binding to form neutrophil–platelet complexes and by phagocytosing activated platelets [27]. While we cannot totally rule out the possibility of contamination of neutrophils by platelet–neutrophil complexes in the LV infarct, platelets were excluded during the cell isolation procedure.

Increased recruitment into the infarct region was demonstrated by a peak in calgranulin B at MI D1 (Fig. 3c) [9, 38, 39]. Further evidence of degranulation was the increased release of matrix metalloproteinase (MMP)-8 and -9 into the infarct region (Fig. 3d, e). MMP-8 and MMP-9 are released into the ECM from neutrophil gelatinase granules to degrade ECM and propagate inflammatory signaling [26]. Overall, the MI D1 neutrophils responded by turning on leukocyte recruitment and stimulating inflammatory signaling through degranulation. Neutrophils at this time reflected the well-characterized response to injury [2].

### MI D3 neutrophil polarization phenotype: similar to D1 cell, along with increased apoptotic signaling, ECM reorganization, and cathepsin activity

**Fig. 4:**
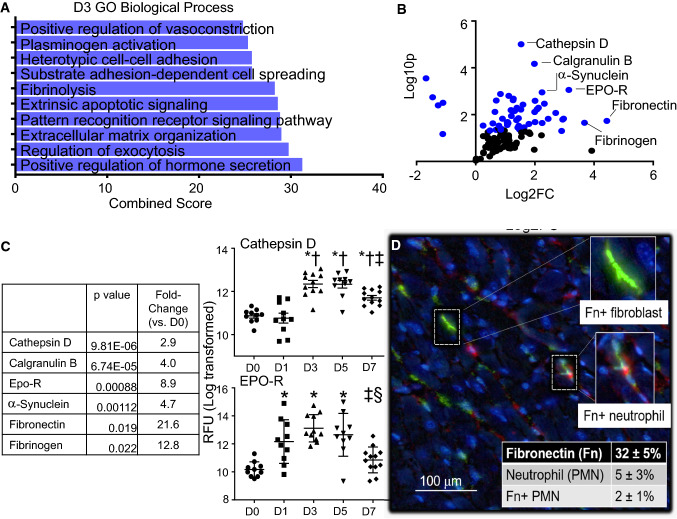
Neutrophil expression profile at day 3 post-MI. **a** GO biological processes enriched in day 3 MI neutrophils. **b** Volcano plot analysis, with downregulated proteins on the left and upregulated proteins on the right. Proteins with *p* values < 0.05 are shown in blue. **c** The highest fold changes at day 3 MI were cathepsin D, calgranulin B, and Epo-R. Time course of cathepsin D and Epo-R protein expression in neutrophils. **d** Multiplex imaging of the infarct region at day 3 MI showing Fn mRNA expression, PMN protein marker expression (Ly6B.2), and colocalization of Fn mRNA in PMN staining. A total of 40% of neutrophils were positive for fibronectin. *p < 0.05 vs. D0, ^†^*p* < 0.05 vs. D1, ^‡^*p* < 0.05 vs. D3, and ^§^*p* < 0.05 vs. D5; and sample sizes are *n* = 10 day 0, *n* = 10 day 1, *n* = 11 day 3, *n* = 10 day 5, and *n* = 12 day 7

Of the GO biological processes, extrinsic apoptotic signaling and fibrinolysis as well as induction of ECM reorganization were upregulated in MI D3 neutrophils (Fig. 4a). Volcano plot analysis and ranking indicated the most prominent increases were in cathepsin D, calgranulin b, erythropoietin receptor (EPO-R), α-synuclein, fibronectin, and fibrinogen to induce intracellular and ECM reorganization (Fig. 4b). Cathepsin D and EPO-R were highest at day 3 (Fig. 4c), concomitant with the initiation of inflammation resolution signaling. Cathepsins are released during degranulation, specifically from ficolin-1 rich granules. Cathepsins are a family of proteases that, like MMPs, can degrade ECM components [37]. They have also been linked to autophagy and have been shown to mitigate the damage of cardiac remodeling [46]. Cathepsin D has been shown to mediate cytochrome C and caspase activity in neutrophil apoptosis, suggesting an intracellular and extracellular role of cathepsins [8].

Because fibronectin increased 22-fold in MI D3 neutrophils compared to D0 cardiac neutrophils, we evaluated whether the neutrophil was the source or recipient of fibronectin protein. By in situ hybridization, fibronectin-positive cells contributed 32% of the total cells within the MI D3 left ventricle infarct region (Fig. 4d). As expected, fibroblasts were positive for fibronectin [32]. The total percentage of neutrophils was 5% of the infarct region. Within the neutrophil pool, 2% of the total infarct area (or 40% of neutrophils) was also positive for fibronectin. Overall, the MI D3 neutrophil showed increased cathepsin activity and apoptotic signaling along with the initiation of ECM production.

### MI D5 neutrophil polarization phenotype: increased ECM reorganization, reduced neutrophil recruitment, and resolution of inflammation

**Fig. 5:**
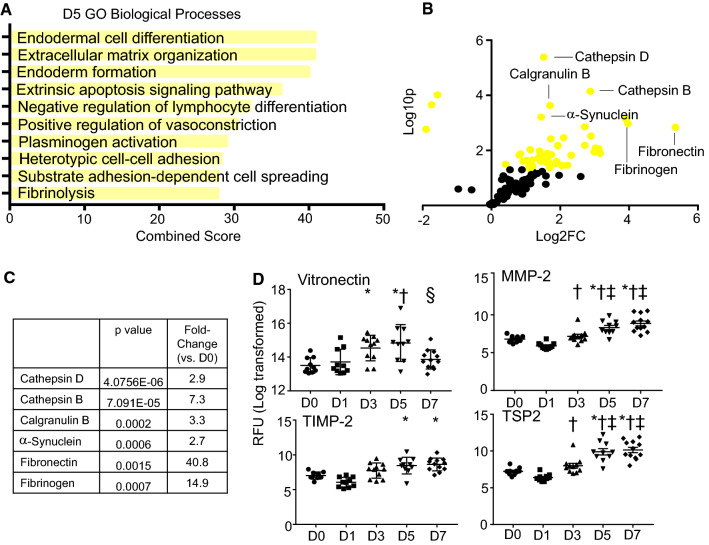
Neutrophil expression profile at day 5 post-MI. **a** GO biological processes enriched in day 5 MI neutrophils. **b** Volcano plot analysis, with downregulated proteins on the left and upregulated proteins on the right. Proteins with *p* values < 0.05 are shown in yellow. **c** The highest fold changes at day 5 MI were Cathepsin D, cathepsin B, and calgranulin B. **d** Time course of vitronectin, MMP-2, TIMP-2, and TSP-2 protein expression in neutrophils. *p < 0.05 vs. D0, ^†^*p* < 0.05 vs. D1, ^‡^*p* < 0.05 vs. D3, and ^§^*p* < 0.05 vs. D5; and sample sizes are *n* = 10 day 0, *n* = 10 day 1, *n* = 11 day 3, *n* = 10 day 5, and *n* = 12 day 7

Of the GO biological processes, there was a pronounced increase in intracellular and ECM reorganization at MI D5 (Fig. 5a). In the volcano plot and by ranking, the most prominent increases were in cathepsins D and B, calgranulin b, α-synuclein, fibrinogen, and fibronectin (Fig. 5b, c). Additional ECM components upregulated at MI D5 were vitronectin, MMP-2, tissue inhibitor of metalloproteinase (TIMP)-2, and thrombospondin-2 (Fig. 5d). Vitronectin modulates neutrophil adhesion and chemotaxis to promote pro-inflammatory responses, as well as delays neutrophil apoptosis [3]. Neutrophil adhesion to vitronectin is enhanced in the presence of chemotactic agonists to accumulate neutrophils at inflammatory sites [24]. While only recently has MMP-2 been attributed to neutrophils, neutrophil expression of TIMP-2 or thrombospondin-2 has not been reported [29].

### MI D7 neutrophil polarization phenotype: ECM synthesis and organization

**Fig. 6:**
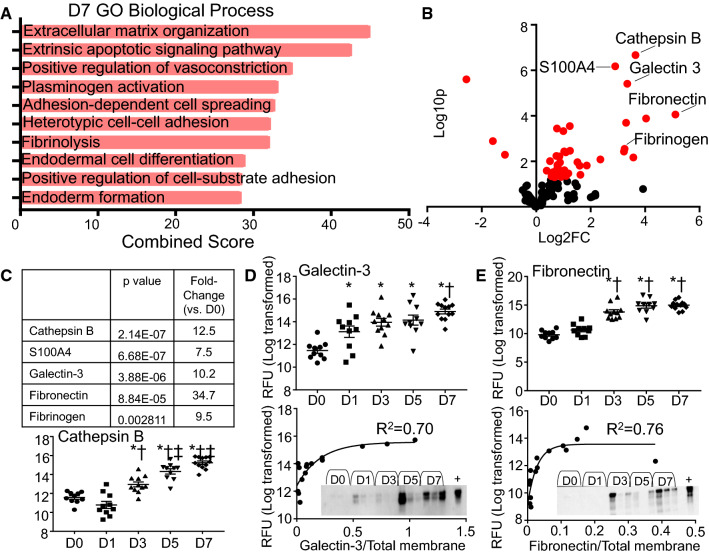
Neutrophil expression profile at day 7 post-MI. **a** GO biological processes enriched in day 7 MI neutrophils. **b** Volcano plot analysis, with downregulated proteins on the left and upregulated proteins on the right. Proteins with *p* values < 0.05 are shown in red. **c** The highest fold changes at day 7 MI were cathepsin B, S100A4, and galectin-3. Time course of cathepsin B, **d** Galectin-3, and **e** fibronectin protein expression in neutrophils by aptamer proteomics (top). Goodness of fit *R*^2^ values were obtained by one-phase association validation analysis for **d** galectin-3 and **e** fibronectin comparing aptamer proteomics in the *y* axis and immunoblotting normalized to total membrane staining in the *x* axis. *p < 0.05 vs. D0, ^†^*p* < 0.05 vs. D1, ^‡^*p* < 0.05 vs. D3, and ^§^*p* < 0.05 vs. D5; and sample sizes are *n* = 10 day 0, *n* = 10 day 1, *n* = 11 day 3, *n* = 10 day 5, and *n* = 12 day 7

The top GO biological process at MI D7 was ECM organization (Fig. 6a). Cathepsin B, fibrinogen, and fibronectin continued to be high, and galectin-3 and S100A4 were also highly expressed (Fig. 6b). Cathepsin B and galectin-3 peaked at MI D7 (Fig. 6c). Validation of the aptamer proteomics results by immunoblotting showed that galectin-3 and fibronectin results were consistent between techniques (Fig. 6d, e). While the numbers of neutrophils are low at day 7, they contribute to the new ECM landscape by promoting ECM reorganization [26].

### Fibronectin stimulates selective neutrophil degranulation

**Fig. 7:**
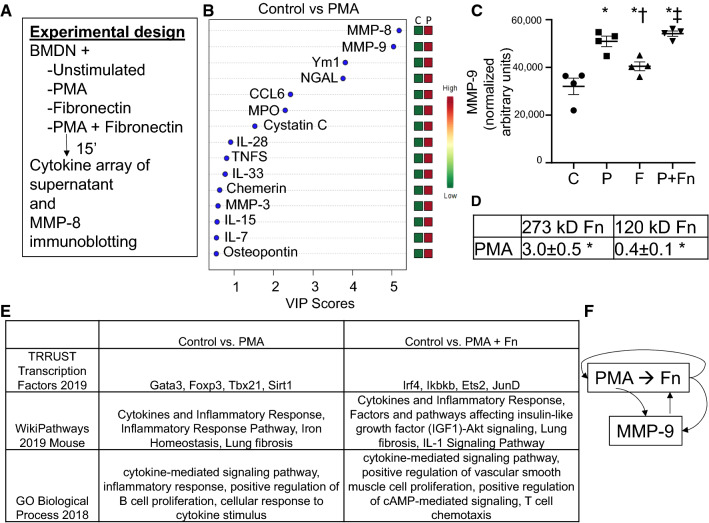
Stimulation of bone marrow-derived neutrophils with fibronectin induces selective degranulation. **a** Experimental design. **b** Variable importance of projection (VIP) scores for control vs PMA stimulated neutrophils. **c** Both PMA and fibronectin stimulated MMP-9 protein expression, which was amplified by dual stimulation with both PMA and fibronectin. **d** PMA stimulated the release of full-length 273 kD fibronectin into the supernatant, while suppressing breakdown of fibronectin to its 120 kD fragment by MMP-9. Values are fold-change vs. control. **e** Enrichr analysis revealed that PMA and fibronectin stimulated differential signaling responses. **f** Mechanistic illustration of how PMA stimulated both the release of fibronectin as well as the release of MMP-9 that degrades fibronectin. *n* = 4 per group; *p < 0.05 vs. Control; ^†^*p* < 0.05 vs. PMA; and ^‡^*p* < 0.05 vs. Fn

To understand the feedback between neutrophils, fibronectin, and MMP-9, we stimulated bone marrow-derived neutrophils with fibronectin and measured degranulation of proteins into the supernatant (Fig. 7a). We focused on MMP-9 because it is a degranulation product, and fibronectin is a known MMP-9 substrate [49]. Stimulation with PMA as a positive control resulted in the release of 69 out of 112 proteins measured compared to the unstimulated control (Fig. 7b). Stimulation with fibronectin alone induced only MMP-9 and neutrophil gelatinase-associated lipocalin (NGAL), providing a mechanism to break down fibronectin and serve as a negative feedback signal. Fibronectin tempered the effects of PMA, resulting in upregulation of 53 (instead of 69) proteins compared to control. PMA or fibronectin alone stimulated the release of MMP-9. In combination, there was an additive effect and MMP-9 levels were amplified compared to either single stimulus (Fig. 7c). Because PMA signals through protein kinase C, the difference in signaling pattern with fibronectin indicates that it does not work through protein kinase C signaling [11].

By immunoblotting examination, PMA stimulation increased the release of fibronectin by neutrophils and reduced fibronectin fragmentation to increase fibronectin availability (Fig. 7d). Thus, MMP-9 was not as effective in breaking down fibronectin when in the presence of a pro-inflammatory stimulus. By Enrichr evaluation of protein–protein interactions, the primary transcription factors induced by PMA were Gata3, Foxp3, Tbx21, and Sirt1. Fibronectin shifted the transcription factor profile to Irf4, Ikbkb, Ets2, and JunD (Fig. 7e). These results indicate that fibronectin selectively degranulates neutrophils and in the presence of a pro-inflammatory stimulus amplified the release of MMP-9 available to degrade fibronectin to its 120 kD fragment (Fig. 7f).

## Discussion

**Fig. 8:**
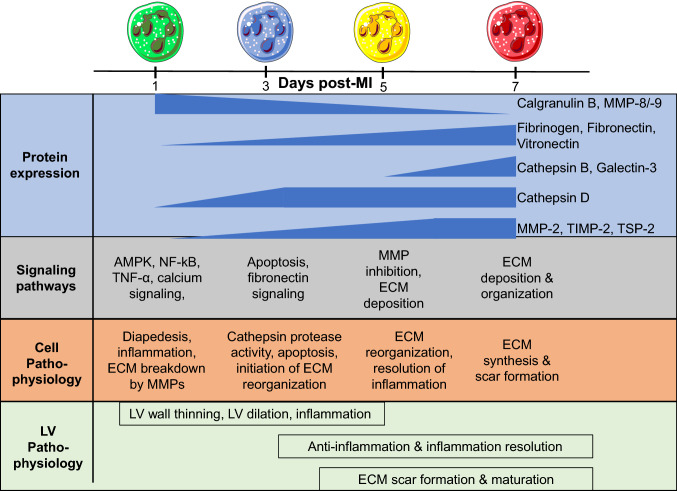
Temporal polarization of neutrophils from day 0 to day 7; highlighting proteins reflective of selective degranulation, protein activity and signaling, and cell and left ventricle (LV) physiology

The goal of this study was to map the continuum of polarization phenotypes in cardiac neutrophils over the first week of MI. The key finding was that cardiac neutrophils undergo continual and distinct proteomic evolution over the first week of MI, comprising differential shifts in protein composition. Day (D)1 MI neutrophils had a high degranulation profile with increased MMP activity. D3 MI neutrophil profiles showed upregulation of apoptosis and induction of ECM organization. D5 MI neutrophils further ramped up their ECM reorganization profile. D7 MI neutrophils had a reparative signature that included expression of fibronectin, galectin-3, and fibrinogen to contribute to scar formation by stimulating ECM reorganization. Figure 8 illustrates the MI neutrophil continuum. Overall, our results indicate that neutrophils selectively degranulate over the MI time course, dependent on their protein profiles as well as the ECM environment in which they reside. MMPs, cathepsins, and ECM proteins (fibronectin, fibrinogen, galectin-3, thrombospondin-2, and vitronectin) were prominent neutrophil degranulation indicators. Of note, fibronectin is a key modulator of degranulation, as it amplified MMP-9 release in the presence of an inflammatory stimulus.

Neutrophils are first responders in the inflammatory process [25]. Early after MI, neutrophils infiltrate infarcted tissue and interact with surrounding endogenous cardiac cells to propagate inflammation by interacting with DAMPs such as high mobility group box 1 protein and heat shock protein 60 produced by injured host tissue [35, 45]. Neutrophils are professional phagocytes as well, engulfing debris and degraded materials. Removal of necrotic tissue is a necessary process for healthy scar formation [12]. Our results indicate that there is a larger pool of D0 resident cardiac neutrophils than previously appreciated. The high engulfment of cardiomyocyte proteins (troponins I and T) indicates that the resident cardiac neutrophils may be performing a surveillance function.

At D3 MI, neutrophils begin to undergo apoptosis. In addition to apoptosis, neutrophils undergo NETosis and can undergo polarization to a phenotype expressing anti-inflammatory factors [26, 36]. The ECM proteins identified may actually help to form the NETosis structure. Interactions between macrophages and apoptotic neutrophils induce an M2 anti-inflammatory phenotype in macrophages to initiate the wound-healing phase of cardiac remodeling [18]. While we know neutrophils and macrophages interact, this study provides novel insight into the neutrophil as an active contributor of ECM production and organization.

The initiation of ECM reorganization occurred concomitantly at D3 MI along with increased production of ECM proteins, fibrinogen and fibronectin. Fibrinogen activates fibroblasts during wound healing and thus may play an important role in remodeling and fibrosis in the heart post-MI [16]. Fibronectin has not been reported to be expressed by neutrophils; our results confirmed that 40% of D3 MI neutrophils expressed fibronectin mRNA. Our study revealed a novel feedback loop between fibronectin and MMP-9, both contributed by the neutrophil that would provide a fine layer of regulation of wound healing within the infarct zone localized to the site of neutrophil entry.

Neutrophils were evolutionarily derived as an immediate means to protect against infection, releasing a large variety of antibacterial agents and destructive enzymes through a multi-granule delivery system [13]. Secondary granules contain neutrophil gelatinase-associated lipocalin, and tertiary (gelatinase) granules contain matrix metalloproteinase (MMP)-8 and MMP-9 [25]. The Borregaard laboratory has recently performed proteome profiling of human neutrophils to catalog granule subsets and identified a new granule type, the ficolin-1 rich granule that contains high levels of cathepsins B, D, H, S, and Z [37]. Our results indicate that this granule is highly released at D3–7 MI. Neutrophil numbers are highest at MI days 1 and 3, with numbers returning toward baseline by day 7 due to macrophage phagocytosis of apoptotic neutrophils to remove them from the LV infarct [28].

Whether the change in protein expression reflects a change in neutrophil subset composition over time (different infiltrating phenotypes) or the day 1 N1 neutrophils develop into pro-resolving day 7 neutrophils remains to be determined. Sorting and analyzing Ly6G+ CD206+ and Ly6G+ CD206− neutrophils separately will be informative, as would single cell and labeling for half-life determination experiments. While neutrophils in circulation mirror some components of tissue neutrophil phenotypes [4, 9], we have shown that circulating neutrophils at day 0 or MI days 1, 3, and 5 were all CD206− [26]. This indicates that CD206+ N2 neutrophils are locally activated in the LV infarct. Our study adds to our current knowledge by revealing distinct neutrophil profiles across the first week of MI.

We are the first to report that neutrophils contribute ECM components. Contribution of ECM components was more prominent in D5–7 MI, correlating with the temporal polarization of neutrophils from N1 to an N2 phenotype. While there are fewer total neutrophils at later times, ECM production is prominent. How neutrophils coordinate with the fibroblasts remains to be revealed; it may be that neutrophils at later time points serve a fine-tuning role to keep the scar localized to the region of the infarct. Fibronectin and fibrinogen are ECM proteins with both structural and signaling properties. Stimulation of neutrophils with fibronectin induced degranulation, and multiplex imaging confirmed neutrophils contain fibronectin mRNA. These results reveal that neutrophils can act in an autocrine manner to induce their own selective degranulation and release the MMPs that shut off further signaling.

In conclusion, neutrophils showed distinct proteomic profiles over the MI time course. Our findings provide novel insights into mechanisms that both regulate and are regulated by neutrophils during MI. Our work indicates that therapeutic strategies for MI remodeling should include consideration for effects on neutrophil subtypes.

## Electronic supplementary material

Below is the link to the electronic supplementary material.

Supplementary material 1 (DOCX 39 kb)

Supplementary material 2 (PPTX 417 kb)

Supplementary material 3 (XLSX 58 kb)

Supplementary material 4 (XLSX 13 kb)

Supplementary material 5 (XLSX 29 kb)
