# Supplementary material for: Neutrophil proteome shifts over the myocardial infarction time continuum
Source: Basic Res Cardiol. 2019 Aug 15;114(5):37. doi: 10.1007/s00395-019-0746-x (PMC6695384; doi:10.1007/s00395-019-0746-x)
Supplement: Supplementary file 1 — Supplementary material 1 (DOCX 39 kb) [file 395_2019_746_MOESM1_ESM.docx]

**Supplemental Methods**

**Animal Use.** All procedures involving mice were approved by the Institutional Animal Care and Use Committee at the University of Mississippi Medical Center. Adult (3-6 month old) C57BL6/J male mice were used for this study. To reduce animal use as recommended by the National Centre for the Replacement, Reﬁnement, and Reduction of Animals in Research,^1^ the neutrophils used in this study were obtained from the same mice used previously for macrophage, fibroblast, and neutrophil isolations and evaluations.^2-5^ Sample sizes are n=10 day 0, n=10 day 1, n=11 day 3, n=10 day 5, and n=12 day 7. For the day samples, the number of cells from pooled mice and the number of cells from individual mice: day 0- 4 pooled and 6 individual; day 1- 3 pooled and 7 individual; day 3- 3 pooled and 8 individual; day 5- 10 individual; and day 7- 4 pooled and 8 individual. We did not observe any differences in results between cells that were from pooled or individual mice.

**Coronary Artery Ligation.** Mice underwent coronary artery ligation surgery to induce permanent MI as described previously and according to the Guidelines for Experimental Models of Ischemia and Infarction.^2-10^ Mice were anesthetized with 2% isoflurane, intubated, and ventilated. The left coronary artery was ligated with 8-0 suture, and MI was confirmed by left ventricle (LV) blanching and ST-segment elevation by EKG. Mice were administered buprenorphine (0.5 mg/kg body weight) immediately before surgery.

**Echocardiography and Necropsy.** LV physiology was measured by echocardiography (Vevo 2100, VisualSonics; Toronto, CA) according to guidelines and as described previously.^2, 8-11^ Mice were anesthetized under 1-2% isoflurane, and both long and short-axis images were obtained. Measurements were taken on the terminal day and were averaged from three cardiac cycles for each mouse. Following imaging, the hearts were removed and the left ventricle (LV) divided into remote and infarct (which included border zone) regions. Each region was separately weighed for infarct area estimation.

**Isolation of LV Infarct Neutrophils.** After digestion in collagenase II (Worthington; Lakewood, NJ) and DNase, a single cell suspension was generated and filtered through a 30 µm separation column. The cell suspension was incubated at 4ºC with a Ly6G-biotin antibody (Miltenyi Biotech, Bergisch Gladbach, Germany, 130-092-332) to collect the neutrophils. The cell pellets were snap frozen and stored at -80°C until use.

**Noise evaluation for the aptamer proteomics dataset.** The noise threshold was calculated for each protein by averaging relative fluorescence units and dividing all day averages by day 0 average set to 1.0. The data were compared to known negative proteins in the dataset- C34 gp41 HIV fragment and HIV-2 Rev (human proteins that should not be present in mouse neutrophils). The C34 gp41 HIV fragment pattern showed a decrease at day 1, increase at days 3 and 5, then decreased at day 7 compared to day 5 (all p<0.05).

For proteins that decreased at day 1, data were kept if the pattern showed a) decrease at day 1 that remained decreased at days 3, 5, and 7; b) decrease at day 1 that went high at day 3 and progressively reduced from days 3 to 5 to 7; or c) decrease at day 1 that progressively increased at days 3, 5, and 7. We considered as noise proteins that went a) down at day 1 then up at days 3 and 5 and back down or no change at day 7 compared to day 5 or b) down at day 1 then up at day 3 and stayed elevated at days 5 and 7.

The data were compared to known positive controls in the dataset, including macrophage mannose receptor (MMR), toll-like receptor 4 (TLR4), periostin (POSTN), matrix metalloproteinase-9 (MMP-9), and tissue inhibitor of metalloproteinase-1 (TIMP-1). Except for MMR, all of these proteins were not detected at levels above the noise pattern and were excluded from the analysis set. All proteins that showed increase at day 1 (fold-change > 1.0) were included. One protein, **glial fibrillary acidic protein (GFAP), was high only at day 7 and was included.**

**The final count was of 1,305 proteins measured, 1,182 (91%) had a noise pattern and were excluded from further analysis. The remaining 123 (9%; Supplemental Table 1) underwent statistical and bioinformatics assessments. Of the 123 proteins, 56 had ANOVA FDR values <0.05 (Supplemental Table 2)**

**Immunoblotting.** For immunoblotting using neutrophil cell pellets, the antibodies used were MMP-8 (1:500, ab81286, abcam, Cambridgem MA), MMP-9 (1:500, AF909, R&D, Minneapolis, MN), galectin-3 (1:500, AF1197, R&D, Minneapolis, MN), and fibronectin (1:5000, AB1954, Millipore, Burlington, MA). Criterion 4-12% polyacrylamide gels (BioRad) were used for electrophoresis, and the trans-blot turbo transfer pack (BioRad) with 0.2 μm nitrocellulose membranes used for transfer. Detection was performed using ECL prime western blotting reagent (GE healthcare). Densitometry was measured by IQ-TL imaging software and normalized to total membrane staining with MemCodeTM Reversible Protein Stain Kit (Thermo Scientific).

**Multiplex Imaging.** MI day 3 LV sections (n=3) fixed in 10% zinc-buffered formalin, paraffin-embedded, and sectioned at 5 µm were used from the mouse heart attack research tool tissue bank.^12^ In situ hybridization was performed with one probe per section using the RNAscope Multiplex Fluorescent Reagent Kit v2 (Advanced Cell Diagnostics, Newark, CA). Samples were hybridized using a probe (Advanced Cell Diagnostics) specific for Fn1 mRNA (1:100); Neutrophils were stained with a neutrophil-specific antibody (1:100) (ab21595, Abcam, Cambridge, UK). Cell nuclei were stained with 4′,6-diamidino-2-phenylindole (DAPI). The probe and antibody were conjugated to the Cy3 and Cy5 fluorophores (Opal 620 and Opal 690, Perkin Elmer, Waltham, MA). Images were acquired at 40× using the Mantra Quantitative Pathology Imaging System (Perkin Elmer), and neutrophil and Fn1 staining were quantified as percent area of the field using inForm cell analysis (Perkin Elmer).

**Supplemental Results**

**Supplemental Table 1.** SomaLogic results for the 123 proteins that passed quality control.

**Supplemental Table 2.** ANOVA values for the 56 proteins with FDR values <0.05.

**Supplemental Figure 1.** Representative protein arrays for unstimulated, phorbol myristate acetate (PMA, 20 nM), fibronectin (100 ng/mL), and PMA + fibronectin stimulated groups. Bone marrow derived neutrophils were isolated and stimulated for 15 min at 37°C. Supernatant (200 μL) were loaded onto the protein arrays. MMP-9

**Supplemental Table 3.** Protein array results, in normalized arbitrary units.

**References**

1. Prescott MJ and Lidster K. Improving quality of science through better animal welfare: the NC3Rs strategy. *Lab Anim (NY)*. 2017;46:152-156.

2. Mouton AJ, DeLeon-Pennell KY, Rivera Gonzalez OJ, Flynn ER, Freeman TC, Saucerman JJ, Garrett MR, Ma Y, Harmancey R and Lindsey ML. Mapping macrophage polarization over the myocardial infarction time continuum. *Basic research in cardiology*. 2018;113:26.

3. Ma Y, Yabluchanskiy A, Iyer RP, Cannon PL, Flynn ER, Jung M, Henry J, Cates CA, Deleon-Pennell KY and Lindsey ML. Temporal neutrophil polarization following myocardial infarction. *Cardiovascular research*. 2016;110:51-61.

4. Mouton AJ, Ma Y, Rivera Gonzalez OJ, Daseke MJ, 2nd, Flynn ER, Freeman TC, Garrett MR, DeLeon-Pennell KY and Lindsey ML. Fibroblast polarization over the myocardial infarction time continuum shifts roles from inflammation to angiogenesis. *Basic research in cardiology*. 2019;114:6.

5. Lindsey ML, Jung M, Yabluchanskiy A, Cannon PL, Iyer RP, Flynn ER, DeLeon-Pennell KY, Valerio FM, Harrison CL, Ripplinger CM, Hall ME and Ma Y. Exogenous CXCL4 infusion inhibits macrophage phagocytosis by limiting CD36 signalling to enhance post-myocardial infarction cardiac dilation and mortality. *Cardiovascular research*. 2019;115:395-408.

6. Lindsey ML, Bolli R, Canty JM, Du XJ, Frangogiannis NG, Frantz S, Gourdie RG, Holmes JW, Jones SP, Kloner R, Lefer DJ, Liao R, Murphy E, Ping P, Przyklenk K, Recchia FA, Schwartz Longacre L, Ripplinger CM, Van Eyk JE and Heusch G. Guidelines for Experimental Models of Myocardial Ischemia and Infarction. *American journal of physiology Heart and circulatory physiology*. 2018.

7. Zamilpa R, Zhang J, Chiao YA, de Castro Bras LE, Halade GV, Ma Y, Hacker SO and Lindsey ML. Cardiac wound healing post-myocardial infarction: a novel method to target extracellular matrix remodeling in the left ventricle. *Methods in molecular biology (Clifton, NJ)*. 2013;1037:313-24.

8. DeLeon-Pennell KY, Iyer RP, Ero OK, Cates CA, Flynn ER, Cannon PL, Jung M, Shannon D, Garrett MR, Buchanan W, Hall ME, Ma Y and Lindsey ML. Periodontal-induced chronic inflammation triggers macrophage secretion of Ccl12 to inhibit fibroblast-mediated cardiac wound healing. *JCI insight*. 2017;2.

9. Jung M, Ma Y, Iyer RP, DeLeon-Pennell KY, Yabluchanskiy A, Garrett MR and Lindsey ML. IL-10 improves cardiac remodeling after myocardial infarction by stimulating M2 macrophage polarization and fibroblast activation. *Basic research in cardiology*. 2017;112:33.

10. Meschiari CA, Jung M, Iyer RP, Yabluchanskiy A, Toba H, Garrett MR and Lindsey ML. Macrophage overexpression of matrix metalloproteinase-9 in aged mice improves diastolic physiology and cardiac wound healing after myocardial infarction. *American journal of physiology Heart and circulatory physiology*. 2018;314:H224-H235.

11. Lindsey ML, Kassiri Z, Virag JAI, de Castro Bras LE and Scherrer-Crosbie M. Guidelines for Measuring Cardiac Physiology in Mice. *American journal of physiology Heart and circulatory physiology*. 2018.

12. DeLeon-Pennell KY, Iyer RP, Ma Y, Yabluchanskiy A, Zamilpa R, Chiao YA, Cannon PL, Kaplan A, Cates CA, Flynn ER, Halade GV, de Castro Bras LE and Lindsey ML. The Mouse Heart Attack Research Tool 1.0 database. *American journal of physiology Heart and circulatory physiology*. 2018;315:H522-h530.
