# Supplementary material for: Neutrophil proteome shifts over the myocardial infarction time continuum
Source: Basic Res Cardiol. 2019 Aug 15;114(5):37. doi: 10.1007/s00395-019-0746-x (PMC6695384; doi:10.1007/s00395-019-0746-x)
Supplement: Supplementary file 2 — Supplementary material 2 (PPTX 417 kb) [file 395_2019_746_MOESM2_ESM.pptx]

## Slide 1
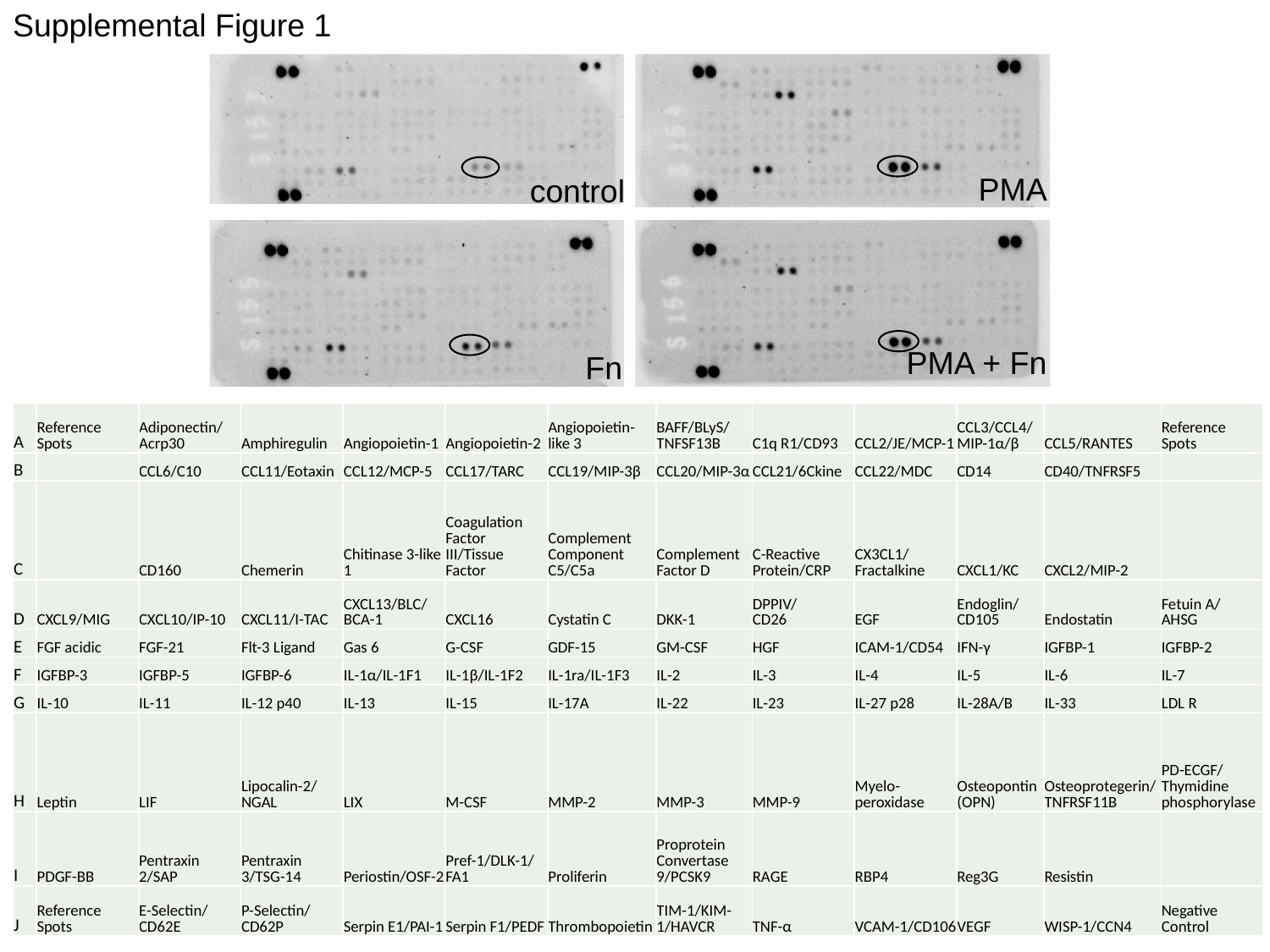

Supplemental Figure 1
PMA
control
PMA + Fn
Fn
| A | Reference Spots | Adiponectin/ Acrp30 | Amphiregulin | Angiopoietin-1 | Angiopoietin-2 | Angiopoietin-like 3 | BAFF/BLyS/ TNFSF13B | C1q R1/CD93 | CCL2/JE/MCP-1 | CCL3/CCL4/ MIP-1α/β | CCL5/RANTES | Reference Spots |
| --- | --- | --- | --- | --- | --- | --- | --- | --- | --- | --- | --- | --- |
| B | | CCL6/C10 | CCL11/Eotaxin | CCL12/MCP-5 | CCL17/TARC | CCL19/MIP-3β | CCL20/MIP-3α | CCL21/6Ckine | CCL22/MDC | CD14 | CD40/TNFRSF5 | |
| C | | CD160 | Chemerin | Chitinase 3-like 1 | Coagulation Factor III/Tissue Factor | Complement Component C5/C5a | Complement Factor D | C-Reactive Protein/CRP | CX3CL1/ Fractalkine | CXCL1/KC | CXCL2/MIP-2 | |
| D | CXCL9/MIG | CXCL10/IP-10 | CXCL11/I-TAC | CXCL13/BLC/ BCA-1 | CXCL16 | Cystatin C | DKK-1 | DPPIV/ CD26 | EGF | Endoglin/ CD105 | Endostatin | Fetuin A/ AHSG |
| E | FGF acidic | FGF-21 | Flt-3 Ligand | Gas 6 | G-CSF | GDF-15 | GM-CSF | HGF | ICAM-1/CD54 | IFN-γ | IGFBP-1 | IGFBP-2 |
| F | IGFBP-3 | IGFBP-5 | IGFBP-6 | IL-1α/IL-1F1 | IL-1β/IL-1F2 | IL-1ra/IL-1F3 | IL-2 | IL-3 | IL-4 | IL-5 | IL-6 | IL-7 |
| G | IL-10 | IL-11 | IL-12 p40 | IL-13 | IL-15 | IL-17A | IL-22 | IL-23 | IL-27 p28 | IL-28A/B | IL-33 | LDL R |
| H | Leptin | LIF | Lipocalin-2/NGAL | LIX | M-CSF | MMP-2 | MMP-3 | MMP-9 | Myelo-peroxidase | Osteopontin (OPN) | Osteoprotegerin/TNFRSF11B | PD-ECGF/ Thymidine phosphorylase |
| I | PDGF-BB | Pentraxin 2/SAP | Pentraxin 3/TSG-14 | Periostin/OSF-2 | Pref-1/DLK-1/FA1 | Proliferin | Proprotein Convertase 9/PCSK9 | RAGE | RBP4 | Reg3G | Resistin | |
| J | Reference Spots | E-Selectin/CD62E | P-Selectin/CD62P | Serpin E1/PAI-1 | Serpin F1/PEDF | Thrombopoietin | TIM-1/KIM-1/HAVCR | TNF-α | VCAM-1/CD106 | VEGF | WISP-1/CCN4 | Negative Control |
